# Update: Ebola Virus Disease Epidemic — West Africa, February 2015

**Published:** 2015-02-27

**Authors:** Incident Management System Ebola Epidemiology Team, CDC; Guinea Interministerial Committee for Response Against the Ebola Virus; World Health Organization; CDC Guinea Response Team; Liberia Ministry of Health and Social Welfare; CDC Liberia Response Team; Sierra Leone Ministry of Health and Sanitation; CDC Sierra Leone Response Team; Viral Special Pathogens Branch, National Center for Emerging and Zoonotic Infectious Diseases, CDC

CDC is assisting ministries of health and working with other organizations to end the ongoing epidemic of Ebola virus disease (Ebola) in West Africa (*1*). The updated data in this report were compiled from situation reports from the Guinea Interministerial Committee for Response Against the Ebola Virus, the Liberia Ministry of Health and Social Welfare, the Sierra Leone Ministry of Health and Sanitation, and the World Health Organization.

According to the latest World Health Organization update on February 18, 2015 (*2*), a total of 23,253 confirmed, probable, and suspected cases of Ebola and 9,380 Ebola-related deaths had been reported as of February 15 from the three West African countries (Guinea, Liberia, and Sierra Leone) where Ebola virus transmission has been widespread and intense. Total case counts include all suspected, probable, and confirmed cases, which are defined similarly by each country (*3*). Because of improvements in surveillance, the number of cases reported in recent weeks might overestimate the number of Ebola cases in some areas because nonconfirmed cases are included in the total case counts. Sierra Leone reported the highest number of laboratory-confirmed cases (8,212), followed by Liberia (3,149) and Guinea (2,727). During the week ending February 14, a daily average of 11 confirmed cases were reported from Sierra Leone, fewer than one from Liberia, and seven from Guinea. The areas with the highest numbers of confirmed cases reported during January 25–February 14 were the Western Area and Port Loko (Sierra Leone) and Forecariah (Guinea) (Figure). Guinea saw an increase in confirmed cases over the past 3 weeks. This might reflect improved surveillance and case reporting because of increased access to previously inaccessible communities.

The latest updates on the ongoing Ebola epidemic in West Africa, including case counts, are available at http://www.cdc.gov/vhf/ebola/outbreaks/2014-west-africa/index.html. The most up-to-date infection control and clinical guidelines for the Ebola epidemic in West Africa are available at http://www.cdc.gov/vhf/ebola/hcp/index.html.

## Figures and Tables

**FIGURE:**
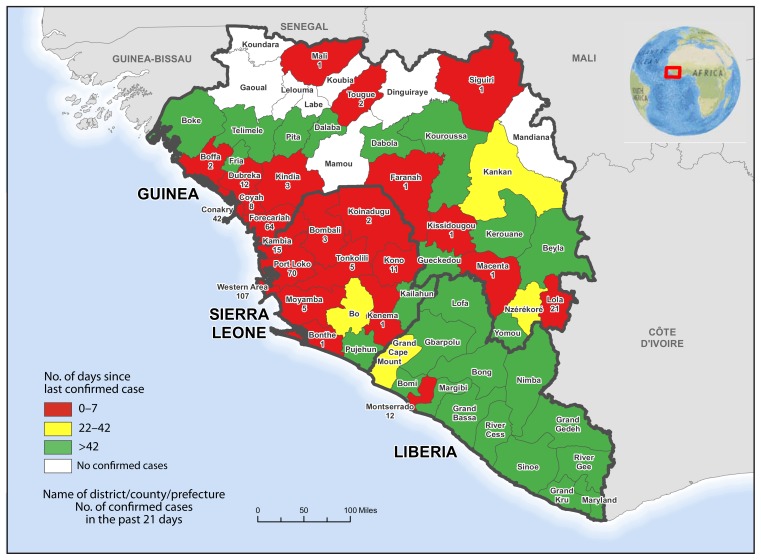
Number of days since last confirmed case of Ebola virus disease and number of confirmed cases in the past 21 days — Guinea, Liberia, and Sierra Leone, January 25–February 14, 2015* **Sources:** Guinea Ministry of Health; Liberia Ministry of Health and Social Welfare; Sierra Leone Ministry of Health and Sanitation; World Health Organization. * Data as of February 14, 2015.
